# Metabolomics of 3D cell co-culture reveals alterations in energy metabolism at the cross-talk of colorectal cancer-adipocytes

**DOI:** 10.3389/fmed.2024.1436866

**Published:** 2024-10-03

**Authors:** Andrea Corazzi Pelosi, Alex Ap. Rosini Silva, Anna Maria Alves Piloto Fernandes, Pedro Paulo Menezes Scariot, Manoela Stahl Parisotto Oliveira, Andreia M. Porcari, Denise Gonçalves Priolli, Leonardo Henrique Dalcheco Messias

**Affiliations:** ^1^Research Group on Technology Applied to Exercise Physiology—GTAFE, Health Sciences Postgraduate Program, São Francisco University, Bragança Paulista, SP, Brazil; ^2^MS4Life Laboratory of Mass Spectrometry, Health Sciences Postgraduate Program, São Francisco University, Bragança Paulista, SP, Brazil; ^3^Coloproctology Service of the Federal University of São Paulo, São Paulo and Faculty of Health Sciences Pitágoras de Codó, Codó, Brazil

**Keywords:** co-culture, metabolomics, neoplasm, rectum, bioinformatics

## Abstract

**Introduction:**

Colorectal cancer (CRC) is the third most incident and the second most lethal malignant tumor. Despite the recognized association between obesity and CRC, further clarification is necessary regarding the lipids that are overexpressed during the development of CRC. In this scenario, the combination of metabolomics and a three-dimensional (3D) co-culture model involving CRC tumor cells and lipids can enhance the knowledge of energy metabolism modifications at the cross-talk between colorectal cancer and adipocytes. This study aimed to screen potential metabolites in the three dimensional (3D) co-culture of CRC and adipocytes by investigating the metabolome composition of this co-culture released into the extracellular space, which is known as the secretome.

**Methods:**

Pre-adipocyte cells (3T3-L1), human colon carcinoma (HT-29), and the 3D co-culture (3T3-L1 + HT-29) were cultured for the secretome obtention. Then, ultra-high-performance liquid chromatography coupled with high-resolution mass spectrometry (LC-HRMS) was employed to analyze the metabolomics of each secretome.

**Results:**

Overall, 3.731 molecules were detected independent of the cell culture. When comparing the three cultures, 105 molecules presented a statistically significant difference in abundance between groups. Among these molecules, 16 were identified, with a particular emphasis on six lipids (PG 20:0, octadecenal, 3-Hydroxytetracosanoyl-CoA, 9,10-dihydroxy-octadecenoic acid, palmitoleic acid, and PA 18:4) and one amino acid derivative (acetylglutamic acid), which presented significant scores during the partial least-squares discriminant analysis (PLS-DA).

**Discussion:**

Although it is too early to determine the possible impact of such molecules in a CRC microenvironment, these results open new avenues for further studies on the energy metabolism at the cross-talk of colorectal cancer adipocytes.

## Introduction

Cancer stands as one of the leading causes of mortality globally, with colorectal cancer (CRC) notably exhibiting high prevalence. Data from the International Agency for Research on Cancer indicate that in 2020 alone, there were 1,931,590 newly diagnosed cases of CRC, making it the third most commonly occurring cancer and the second most lethal (1).

As CRC is largely asymptomatic in the early stages, many patients are diagnosed at advanced stages (2). Implementing a screening program is essential to reduce CRC incidence and mortality rates (3, 4). Generally, the molecules used to predict the prognosis and therapeutic responses, such as the carcinoembryonic antigen (CEA) and carbohydrate antigen 19-9 (CA19-9), exhibit relatively low sensitivity and specificity; therefore, there is a necessity for molecular signatures to identify patients in the early stages of CRC (4). This premise has gained strength in the scientific and medical community given that over the past 30 years, the incidence of early-onset CRC (< 50 years old) has been increasing (5).

Obesity is an equally important health concern, growing in pandemic proportions. Obesity is a state of low-grade inflammation, with lipid mediators acting as potent signaling molecules that may contribute to metabolism changes and lead to the development of CRC (6). Despite epidemiological data supporting an association between obesity and CRC, many questions remain unanswered, including whether we should screen obese patients earlier and how it could be done (7). Further clarifications are also required regarding which lipids are overexpressed during the development of CRC.

In this context, metabolomics has been employed to discover tumor biomarkers for several cancers, aiding in diagnosis, treatment, prognosis, and prevention (8–12). Through the measurement of metabolites in biofluids and/or tissue samples, this technique identifies a wide range of small potential metabolites involved in various biological pathways (13–15). Given the metabolomics' relevance in identifying new biomarkers, it is feasible to investigate the cross-talk between CRC and the metabolome profile using this technique. For this purpose, robust approaches integrating CRC and metabolites at the cellular level are essential, such as the three-dimensional (3D) cell co-culture model. The 3D co-culture model has promoted new horizons in cancer research, particularly in understanding the cancer environment, progression, and immunotherapies (16–18). However, the application of this technique in combination with metabolomics to discover new molecular signatures in the CRC-adipocyte binomial is still incipient.

Therefore, this study aimed to screen potential metabolites in the 3D co-culture of CRC and adipocytes by investigating the metabolite profile when these cancer cells are co-cultured with adipocytes.

## Methods

### Experimental design and protocol registration

**Figure 1:**
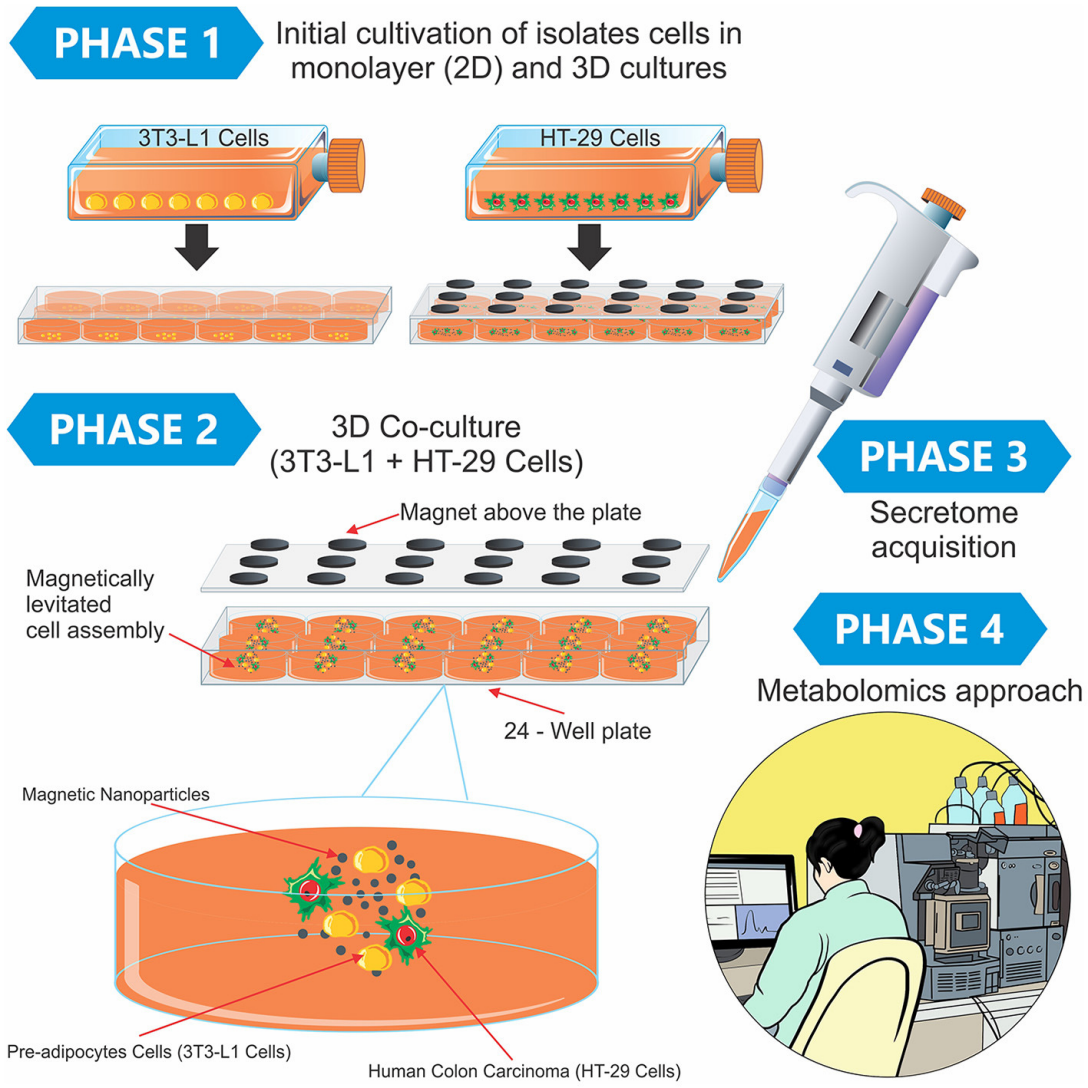
Experimental design containing the phases of the study and a didactic illustration of the magnetic 3D cell culture. In Phase 1, the 3T3-L1 and HT-29 cells were separately cultured using the two-dimensional model in monolayer (2D) and 3D cultures. After this initial preparation, both cells (3T3-L1 + HT-29) were mixed in the 3D co-culture, in which the cells grew in spheroid geometry (Phase 2). Finally, the secretome was obtained from different cultures (Phase 3), and the metabolomics approach was conducted in Phase 4.

The protocol details for the secretome investigation of the tumor 3D co-culture model and the 2D culture model are available at doi.org/10.17504/protocols.io.b24vqgw6. Briefly, pre-adipocyte cells (3T3-L1), human colon carcinoma (HT-29), and the co-culture (3T3-L1 + HT-29) were cultured for the secretome obtention. Finally, high-performance liquid chromatography coupled with high-resolution mass spectrometry (LC-HRMS) was employed to analyze the metabolomics of each secretome (Figure 1).

### 2D cell culture

The human colon carcinoma (HT-29) and pre-adipocyte cells (3T3-L1) were obtained from the Rio de Janeiro Cell Bank (BCRJ). Dulbecco's modified Eagle medium (DMEM) was supplemented with 100 mM of sodium pyruvate, 10% (v/v) of fetal bovine serum (FBS), and 1% of two antibiotics, namely, penicillin (100 U·ml^−1^) and streptomycin (10 mg·ml^−1^).

The cells were cultured in a humidified chamber with 5% (v/v) CO_2_ at 37°C and then incubated with 3 ml of trypsin-ethylenediaminetetraacetic acid (EDTA) 0.25% (v/v) at 37°C for 3 min to allow cell disaggregation and propagation. The DMEM plus 10% FBS was used to inactivate the trypsin-EDTA. The cell pellet was then transferred to a new 75 cm^3^ flask (T75) containing 10 ml of the DMEM. Cell viability was determined in a Neubauer chamber through trypan blue staining.

### 3D cell culture and co-culture

The 3D cell co-culture was conducted by magnetic levitation using the Bio-Assembler™ system in the n3D Biosciences 24-well configuration (19). The magnetic nanoparticles were prepared by removing them from the refrigerator and thawing them at room temperature (20–25°C) for 15 min.

The HT-29 cells were cultured in a monolayer culture in a T75 flask; then, cell viability (>75%) was determined in a Neubauer chamber, when the cells reached 80%−90% confluence. Subsequently, 1 μL per 10,000 cells of the magnetic nanoparticles (Nanoshuttle™- PL, Greiner, São Paulo, Brazil) was added to the single-cell suspension flask, and it was homogenized and centrifuged three times at 1.500 rpm for 5 min. After centrifugation, the cells were resuspended and added to a cell-repellent 24-well plate with enough solution to reach 7.5 × 10^3^ cells. Furthermore, 250 μL of the previously described DMEM (supplemented) was added to achieve a volume of 250 μL/well. A magnetic coupling driver was placed under the plate for 5 h and incubated in the humidified chamber with 5% (v/v) of CO_2_ at 37°C. The plate was sealed, the levitation drive was placed atop the intermediate lid to levitate the cells, and the magnetic coupling drive was maintained for 7 days. Field microscopy was used to verify the cohesion of the structures formed, and the culture medium was collected whenever the exchange became necessary.

The 3T3-L1 cells (600 μL) were cultured in a monolayer to reach 90% confluence and cell viability (>75%). Then, the cells were incubated in the supplemented medium in a repellent hanging drop plate at 37°C with 5% (v/v) CO_2_ and 95% humidity. The cultured cells were monitored until the aggregates were formed and differentiated into adipocytes. Finally, the 3T3-L1 spheroid suspension was added to each well of the cell-repellent HT-29 cell plate using a magnetic pen 21 days after the beginning of the HT-29 spheroid formation, and this setup was maintained for 7 days. An illustration of the different phases of cell culturing is shown in Figure 1.

### Sample extraction

The culture medium (200 μL) of each well was pipetted into a microtube. Subsequently, 50 μL of iced isopropanol was added and stored at −20°C overnight. Each sample was prepared in triplicate. In addition, a pooled quality control (QC) sample was prepared by aliquoting 20 μL of each sample and pooling together in a microtube to generate a QC sample containing the chemical composition representative of the sample set. Then, the QC pool was homogenized and splited into different vials, and prepared following the same extraction protocol. These samples were used to check the system's suitability before batch analysis. Blank samples (*n* = 3) were also prepared employing the same protocol, using the culture medium without adding any cell or culture. The samples were randomly organized for the extraction process. They were centrifuged at 12,880 × *g* at 4°C for 10 min. An aliquot (150 μL) of each sample was then removed and dried under a nitrogen gas flow. The samples were then resuspended in 150 μL of a solution composed of the internal standard p-Fluoro-DL-phenylalanine at a concentration of 200 μM in methanol.

### Secretome metabolomic analysis

The metabolites were analyzed using an ACQUITY UPLC system coupled with a XEVO G2-XS (QTOF) quadruple time-of-flight mass spectrometer (Waters, Manchester, UK), which was equipped with an electrospray ionization (ESI) source and operated in the negative ionization mode. Chromatographic analysis was performed using an ACQUITY UPLC^®^ CSH C18 column (C18, 2.1 mm × 100 mm × 1.7 μm, Waters) with mobile phase A composed of water (H_2_O) with 0.1% formic acid and mobile phase B composed of acetonitrile (ACN). The flow rate was set at 0.4 ml·min^−1^. Initially, the column was conditioned with 10% of solution B for 2 min. Then the concentration of solution was increased to 90% of B over the next 5 min, where it remained for 2 min. After this period, the concentration of solution B was reduced back to 10% in 2.0 min, equilibrating the column for the next injection for another 2.0 min. The total execution time was 13 min. The injection volume for each column and ionization mode was 2 μL.

The mass spectrometer was operated in the MS^E^ mode with an *m/z* range of 50–1,200 Da and an acquisition time of 0.5 s per scan. For the MS^E^ analysis, the device was set to increase the voltage gradually, starting at 6 V for low collision energy and ramping up to 20–50 V for high collision energy. Other parameters were as follows: source temperature = 140°C, desolvation temperature = 550°C, desolvation gas flow = 900 L·h^−1^, capillary voltage = 2.5 kV, and cone voltage = 40 V. Leucine enkephalin (molecular weight = 555.62; 200 pg·L^−1^ in 1:1 ACN: H_2_O, v/v) was used as a lock mass for mass accuracy, and 0.5 mM of sodium formate solution was used for calibration.

### Data pre-processing and identification of metabolites

The LC–MS raw files were processed using the Progenesis™ QI software v2.4 (Nonlinear Dynamics, Newcastle, United Kingdom), which allowed the selection of possible adducts, peak alignments, deconvolution, and compound annotations based on the MS^E^ experiments (12). The adducts, [M-H]^−^, [M+Cl]^−^, [M-H_2_O-H]^−^, and [M+FA-H]^−^, were considered for the negative acquisition mode. Progenesis QI generated an intensity table of the ions, labeled according to their retention time and nominal masses, called features, as a function of their intensity (areas of the extracted ion chromatogram) for each sample.

The identification of the metabolites was based on the MS^E^ experiments. Due to the low and high energy acquisition, in the same spectrum, we acquired information about precursor ions (lower energies; mass error ≤ 5 *ppm*) and their fragments (higher energies; tolerance of ≤ 10 *ppm*). Fragmentation score, mass accuracy, mass error, and isotopic similarity were evaluated for the annotated molecules. To ensure compatibility between Progenesis QI data and external structured data file (SDF)-based spectra libraries, we used an in-house software package named “SDF2PQI” to increase the number of fragment matches (20). SDF2PQI has been recently detailed elsewhere and is freely available as open-source software. External SDF-based spectra libraries were used, such as LipidMaps (http://www.lipidmaps.org/), the Human Metabolome Database (http://www.hmdb.ca/metabolites), and the MoNA—MassBank of North America (https://mona.fiehnlab.ucdavis.edu/).

### Statistical analyses

The data were analyzed on the MetaboAnalyst platform (https://www.metaboanalyst.ca/). The datasets were uploaded and filtered based on the relative standard deviation (RSD) for the intra-batch QC samples, and the analytes found with RSD >30% were not considered for the statistical analysis. Normalization, transformation, and scaling were conducted using the mean, log base 10, and Pareto approaches, respectively. Principal component analysis (PCA) was employed to check the analytical quality. Initially, the molecules detected, but not yet identified, were compared by one-way analysis of variance (ANOVA), with the molecules considered dependent variables and the cultured cells factorials. When a significant difference was identified by one-way ANOVA, the molecule was selected for identification. The identified molecules were again entered into the MetaboAnalyst platform and normalized, transformed, and scaled following the same steps described previously. The partial least-squares discriminant analysis (PLS-DA) discriminated the cultured cells based on the identified molecules. Cross-validation was performed using the leave-one-out cross-validation (LOOCV) method to ensure the reliability of the model, and variable in projection (VIP) scores were checked for further analysis. Molecules with a VIP score of ≥1 were compared by the one-way ANOVA (cultured cells as independent factors), followed by Tukey's *post-hoc* test. In all cases, statistical significance was established at 5%.

## Results

**Figure 2:**
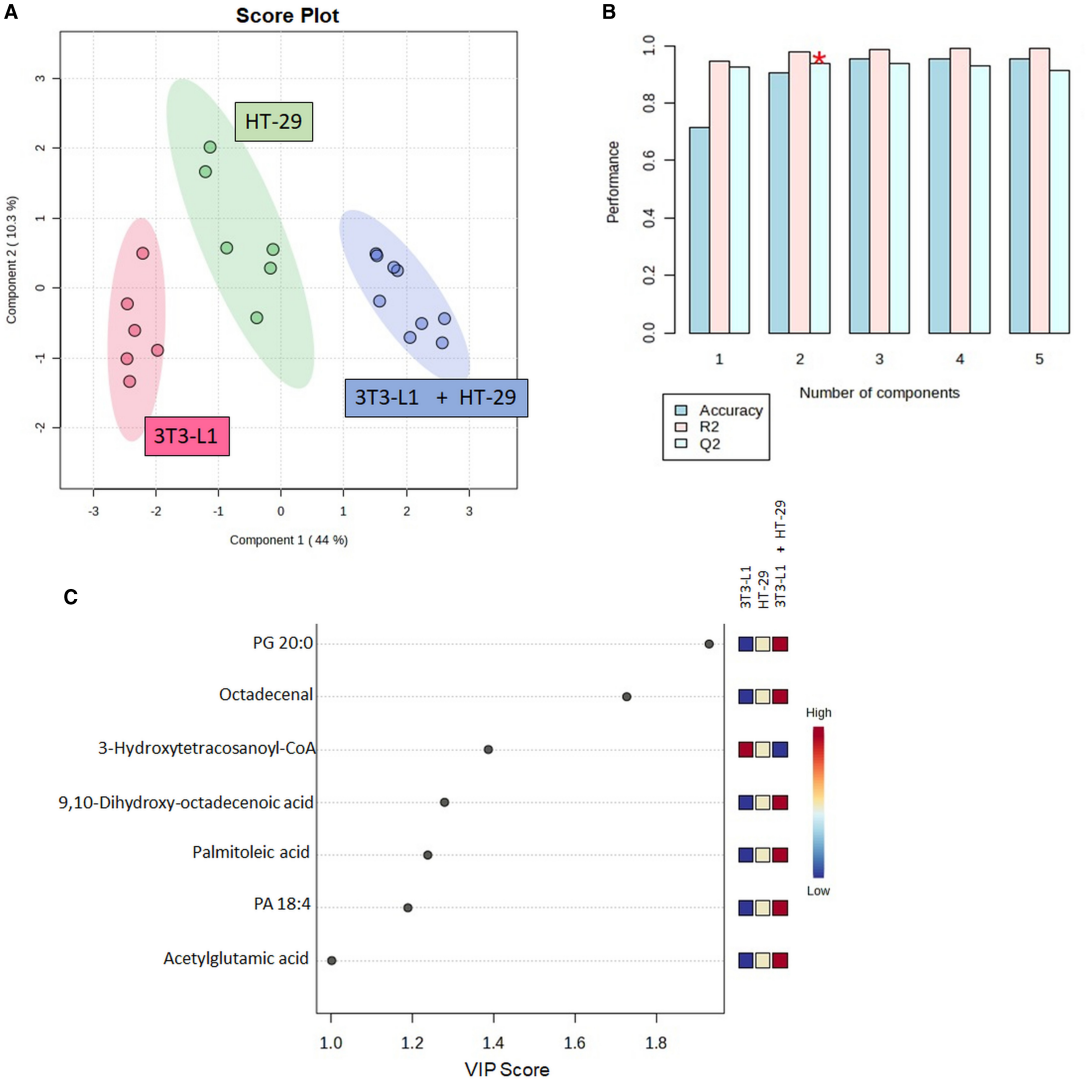
Partial least squares-discriminant analysis (PLS-DA) using the identified molecules in the cell's culture; **(A)** Score plot between components 1 and 2; **(B)** PLS-DA cross-validation based on the leave one out cross-validation (LOOCV) method; **(C)** Molecules with variable in projection (VIP) scores ≥1. CoA, Coenzyme A; PA, Phosphatidic Acid; PG, Phosphatidylglycerol. ^*^Indicates the model's significant predictive ability.

From the raw LC–MS data, 3,731 ions were detected. The analytical quality and the reproducibility of LC-HRMS can be observed by the clustering of the QC group in the PCA score plot (Supplementary Figure 1). The selection of features was based on the one-way ANOVA (*p* < 0.05), with 105 features indicated as differential ones (Supplementary Table 1). From these features, 16 were identified. Table 1 presents the number of molecules with any statistical effect pointed out by the one-way ANOVA. The PLS-DA successfully discriminated the cultured cells (Figure 2A), with components 1 and 2 explaining 44.0 and 10.3% of the variance, respectively. The cross-validation of this model was high regardless of the component (Figure 2B).

**Table 1:** Molecules identified in the cell cultures with any statistical effect found by one-way ANOVA.

***m*/*z* measured**	**Rt (min)**	**Adducts**	**Putative identification**	**Molecular formula**	**Mass error (ppm)**
1,178.4592	0.53	M+FA-H	3-Hydroxytetracosanoyl-CoA	C_45_H_82_N_7_O_18_P_3_S	−3.57
489.1880	4.33	M+FA-H	PA 18:4	C_21_H_33_O_8_P	−3.31
311.2588	7.47	M+FA-H	Octadecenal	C_18_H_34_O	−1.32
253.2161	8.52	M-H	Palmitoleic acid	C_16_H_30_O_2_	−4.60
313.2373	9.07	M-H	9,10-Dihydroxy-octadecenoic acid	C_18_H_34_O_4_	−3.72
599.3184	10.94	M+FA-H	PG 20:0	C_26_H_51_O_10_P	−3.15
170.0450	0.58	M-H_2_O-H	Acetylglutamic acid	C_7_H_11_NO_5_	−4.68
508.0713	1.16	M+FA-H	Adenylsuccinic acid	C_14_H_18_N_5_O_11_P	−2.11
335.0775	0.87	M-H_2_O-H	Chlorogenic acid	C_16_H_18_O_9_	0.84
632.3587	11.01	M+FA-H	PC 22:3	C_30_H_54_NO_8_P	3.02
84.0456	0.77	M-H_2_O-H	3-Aminoisobutanoic acid	C_4_H_9_NO_2_	0.81
677.6097	0.54	M-H	DG 40:1	C_43_H_82_O_5_	1.11
267.0906	4.58	M+Cl	Melatonin	C_13_H_16_N_2_O_2_	−0.12
485.2086	9.25	M+Cl	PA 18:1	C_21_H_39_O_8_P	2.03
288.0393	1.63	M-H_2_O-H	2′-Deoxycytidine 5′-monophosphate	C_9_H_14_N_3_O_7_P	0.70
267.0717	0.77	M-H	3-Deoxy-D-glycero-D-galacto-2-nonulosonic acid	C_9_H_16_O_9_	−1.60

Rt, retention time; VIP, variable in projection; CoA, Coenzyme A; PA, Phosphatidic Acid; PC, Phosphatidylcholine; PG, Phosphatidylglycerol; DG, Diacylglycerol.

**Figure 3:**
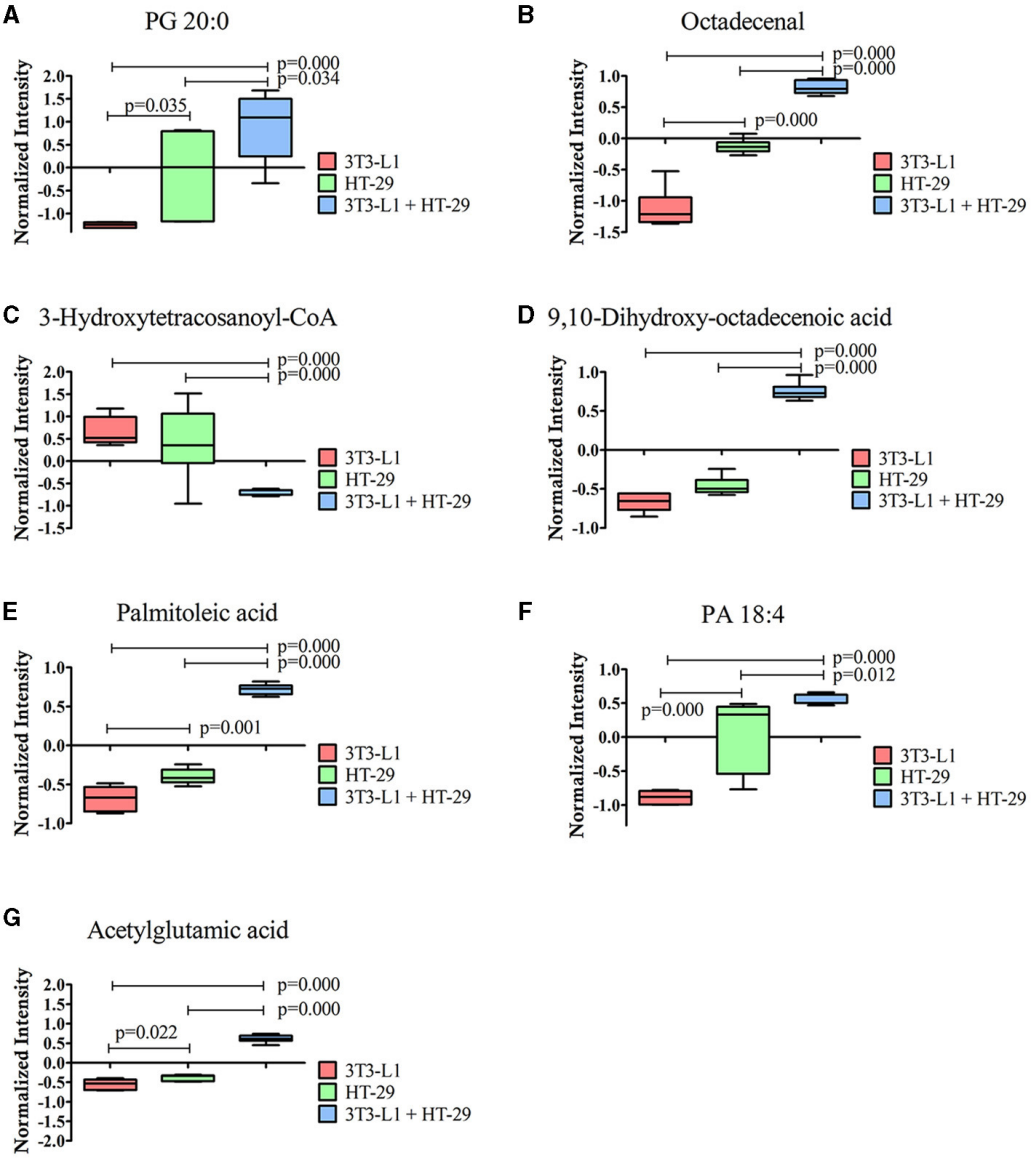
Molecules significantly different between the three-dimensional co-culture (3T3-L1 + HT-29) and the two-dimensional cell's culture (3T3-L1 or HT-29) indicated by one-way ANOVA; *p* ≤ 0.05. **(A)** PG 20:0; **(B)** Octadecenal; **(C)** 3-Hydroxytetracosanoyl-CoA; **(D)** 9,10-dihydroxy-octadecenoic acid; **(E)** Palmitoleic acid; **(F)** PA 18:4; **(G)** acetylglutamic acid. CoA, Coenzyme A; PA, Phosphatidic Acid; PG, Phosphatidylglycerol.

Furthermore, seven out of the 16 identified molecules presented a VIP score of ≥1 (Figure 2C). These molecules were again compared by the one-way ANOVA, as shown in Figure 3. A significant effect of the cultured cell was identified for these molecules. Six molecules were higher in the co-culture compared to 3T3-L1 or HT-29 (PG 20:0, octadecenal, 9,10-dihydroxy-octadecenoic acid, palmitoleic acid, PA 18:4, and acetylglutamic acid), while one (3-hydroxytetracosanoyl-CoA) presented the opposite trend. Apart from acetylglutamic acid, which is an amino acid derivative, the remaining molecules were lipids.

## Discussion

This study unveiled relevant molecular signatures within the intricate interplay between CRC and adipocytes. Through 3D co-culturing, CRC cells with adipocytes, six lipids or lipid-like molecules, and one amino acid derivative were effectively distinguished.

Although 3D culturing is a promising model due to its ability to mimic *in vivo* natural systems (21, 22), the metabolome is the closest link to the phenotype (23). Thus, the combination of these techniques in our experimental design represents a pivotal milestone, paving the way for future clinical investigations in this domain.

Due to the complex and dynamic relationship, the cross-talk between CRC and adipocytes has garnered increasing attention in cancer research (6, 24, 25). Cellular processes, such as signaling pathways, energy storage, and cell membrane composition, are dependent on lipids, including cholesterol derivatives, fatty acids, and phospholipids. Most importantly, alterations in lipid metabolism have been suggested as factors that influence the pathogenesis and progression of tumor growth, metastasis, and response to therapy (26). It would be premature to conclude that the six lipids that exhibit a VIP score of >1 and experience significant modulation in co-culture settings serve as markers of tumor progression or metastatic processes. These lipids play distinct roles in adipocytes or cancer cells.

PG 20:0 refers to phosphatidylglycerol, a lipid found in cellular membranes, as a minor component in comparison to other phospholipids, such as phosphatidylcholine, comprising < 1% (27). However, its involvement in the composition of cancer cell membranes cannot be disregarded. Since phosphatidylglycerol plays a role in the formation of viral envelopes, deviations in its levels may be manifested in virus-associated malignancies, such as cervical cancer (28). According to Szlasa et al. (29), such variations may increase viral replication efficiency and promote cellular neoplasia. Restricted to CRC, phosphatidylglycerol, along with other minor phospholipids, was capable of discriminating malignant from non-malignant human colon specimens (30), but further differentiation regarding the carbon atoms bound to fatty acids was not provided. Furthermore, Shen et al. (31) proposed that PG 34:0 and other molecules have a promising diagnostic potential for distinguishing patients with CRC from healthy individuals. While the co-culture expressed a PG with a distinct fatty acid composition (i.e., 20:0), it is conceivable to suggest this lipid as a potential molecule in the interaction between CRC and adipocytes. Still, further investigation into the precise fatty acid composition of phosphatidylglycerol molecules and their impact on CRC is warranted.

PA 18:4 is a phosphatidic acid that belongs to the main class of glycerophosphoglycerols. Phosphatidic acid comprises 1%−2% of phospholipids in mammalian cell membranes (27). Among the molecules mentioned in this investigation, this acid emerges as potentially having the strongest correlation with the oncogenic processes (32, 33). Although numerous mechanisms underlying phosphatidic acid signaling have been elucidated, it is believed that the mammalian target of rapamycin serves as one of the targets of this lipid in cancer cells (34–36). Direct inferences between CRC and phosphatidic acid have been suggested. For example, cyclic phosphatidic acid, generated from phosphatidic acid by phospholipase D, attenuated CRC cell proliferation and survival by suppressing the peroxisome proliferator-activated receptor γ (37). In addition, it has been suggested that phosphatidic acid facilitates the coalescence of contacting lipid droplets (38), which in turn has been associated with the lifespan of cancer cells (39). Furthermore, in a study, it was observed that lipid droplets contribute to chemoresistance in CRC, although the biogenesis of the droplets was mediated by lysophosphatidylcholine acyltransferase-2 (40). Unfortunately, we could not conclusively establish a direct association between the elevated levels of phosphatidic acid in the co-culture and the aforementioned mechanisms. Nonetheless, these findings support the notion that this lipid is involved in processes pertinent to CRC.

Another lipid highlighted in the three-dimensional co-culture was palmitoleic acid, which is a monounsaturated free fatty acid that regulates metabolic responses between tissues and is often referred to as a lipokine (41). Blood concentrations of this acid showed an inverse correlation with the overall cancer risk (42); however, contrasting conclusions were observed for breast cancer (43) and prostate cancer (44), with the latter being assessed in tissue samples. Regarding the gastrointestinal tract, the serum concentration of palmitoleic acid has been proposed as a promising biomarker for inflammatory bowel diseases, such as Crohn's disease (45). However, this lipid was significantly reduced in the CRC tissue, showing decreases ranging from 20 to 50% compared to the adjacent normal tissue (46). Although our co-culture exhibited elevated concentrations of palmitoleic acid compared to the isolated cultures, we refrain from asserting any direct linkage between this lipid and CRC development or oncogenic mechanisms, particularly in light of the conflicting findings presented in the referenced studies. Indeed, research published over four decades ago demonstrated that supplementation with palmitoleic acid extended the survival duration of mice bearing an Ehrlich ascites tumor (47). However, additional investigations from this standpoint are imperative to elucidate its broader implications and potential therapeutic avenues.

**Figure 4:**
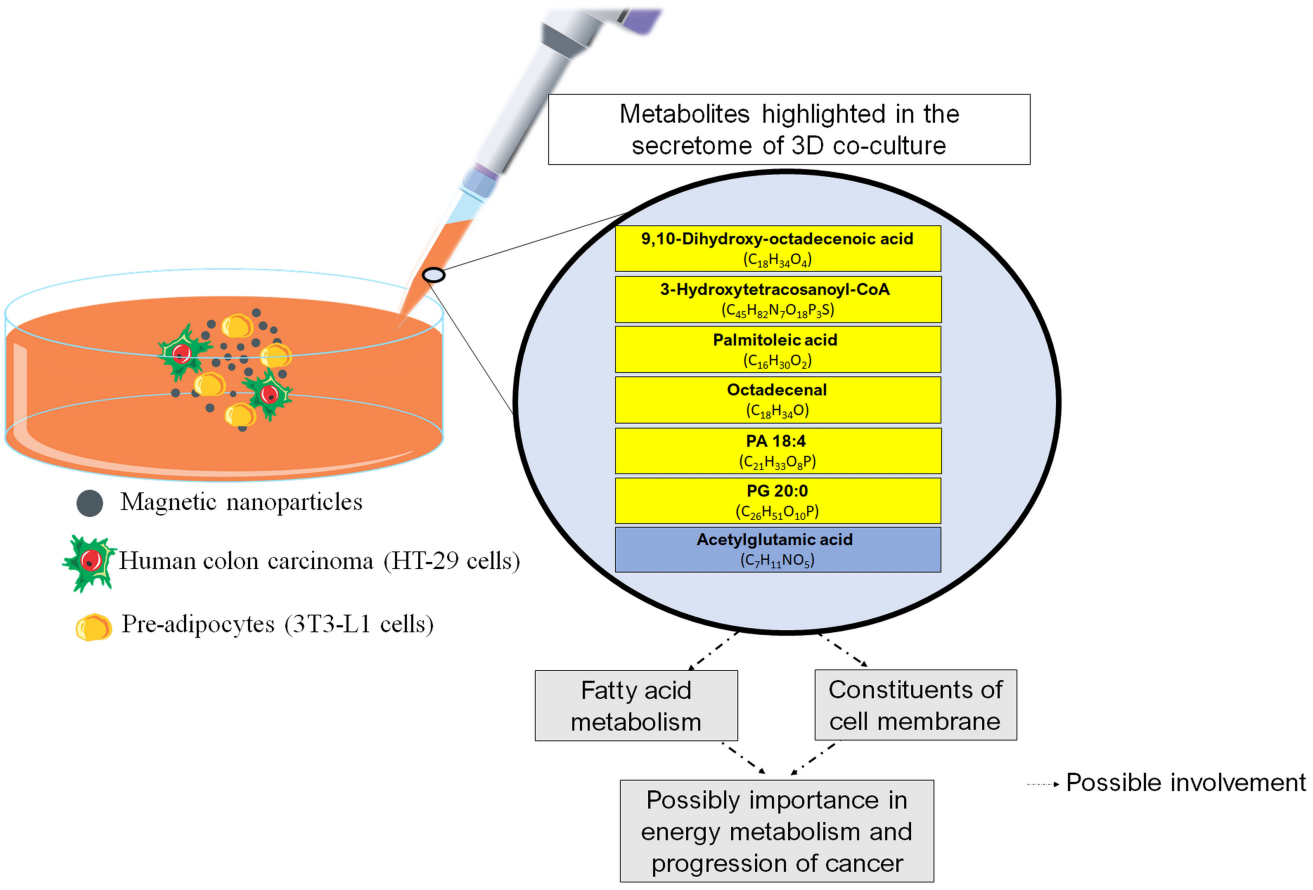
Main findings of the study and possible relationships with physiological aspects. The boxes in yellow refer to the lipids. The box in blue refers to the amino acid. CoA, Coenzyme A; PA, Phosphatidic Acid; PG, Phosphatidylglycerol.

No strong associations between the remaining molecules (octadecenal, dihydroxy-octadecenoic acid, hydroxytetracosanoyl-CoA, and acetylglutamic acid) and CRC were found. Octadecenal is a class of fatty acids found in vegetable oils that is synthesized in the body by the desaturation of stearic acid, while dihydroxy-octadecenoic acid is a polyunsaturated fatty acid derived from linoleic acid oxidation. 3-Hydroxytetracosanoyl-CoA falls within the category of organic compounds termed very-long-chain (3R)-3-hydroxyacyl-CoAs, which are located at the cell's membrane. Notably, among the molecules examined, 3-Hydroxytetracosanoyl-CoA exhibited a distinct outcome within the co-culture setting, with a reduced concentration when compared to the HT-29 and 3T3-L1 cells. We have no data to explain this phenomenon, but we speculate that the reduction may be due to a counter-regulatory response to prevent exacerbated production of acetyl-CoA (48). There is evidence suggesting that acetyl-CoA production (provided by fatty acid oxidation) is an important factor in tumors (49). Acetyl-CoA can be used in the TCA cycle for energy production or in the biosynthesis of cellular membranes for cell proliferation. However, acetylglutamic acid was the only amino acid derivative highlighted in the three-dimensional co-culture. Interestingly, acetylglutamic acid is essential for liver ureagenesis in mammals, and lower serum urea levels are associated with a higher CRC risk (50). These observations lead us to propose that urea metabolism plays an important role in a co-culture (3T3-L1 + HT-29). To discuss the relevant findings, we have outlined the key elements found in the 3D co-culture in Figure 4.

This study has certain limitations. Unfortunately, we were unable to make strong inferences about the physiological mechanisms that overexpressed the lipids or the acetylglutamic acid and colorectal cancer in the co-culture exert. It is unclear whether the increase or reduction in these molecules, such as 3-Hydroxytetracosanoyl-CoA, is associated with pro- or anti-tumor responses. As discussed, although some of the lipids mentioned in this study have implications for the oncogenic process and CRC, the studies cited were cautious in drawing conclusions of this type. Given our experimental design, it is crucial to proceed with caution. To ensure that the insights gained in our study are accurately applicable to real-world biological systems, further research involving animal and human models is essential. This strategy will facilitate the translation of our *in vitro* findings to *in vivo* contexts, allowing us to validate and extend our results in more complex biological environments. Such translational studies are necessary to assess the relevance and efficacy of our model observations in living organisms, ultimately bridging the gap between preclinical findings and clinical applications.

It is also important to emphasize that the discussion about the physiological mechanisms is beyond the objectives of this study. However, Supplementary Table 2 presents some physiological roles of the molecules highlighted in this study. Equally important, the results obtained in this study should be interpreted in accordance with the cell lines used to establish the co-culture. Although HT-29 is largely adopted for colorectal studies including CRC (51), further reports using other CRC cell lines in a 3D co-culture model with pre-adipocytes may determine whether the molecules observed in this study are also present in these cell cultures.

Despite the identified limitations, this study is a first step in exploring new molecular signatures associated with the cross-talk between CRC and adipocytes using a cell co-culture. Another strength of our study is that it was conducted using a method in which cells were levitated off the bottom by a magnet above the plate. This method, known as magnetic 3D cell culturing has been shown to be the most promising model, and this is due to its ability to more closely mimic natural *in vivo* systems (21, 22). The application of magnetic 3D cell culturing is still limited, but this method will certainly provide valuable information and applications in clinical, nutritional, and physiological experimental designs. To the best of our knowledge, our experimental design and outcomes are pioneering in the field of cancer research. Based on the findings of this study, future experimental designs can be developed to further explore the physiological mechanisms that the association between CRC and adipocytes promotes, especially in relation to the lipids found in this study.

## Conclusion

The 3D cell co-culture between human colon carcinoma and pre-adipocyte cells together with the metabolomic analysis revealed six lipids and one amino acid derivative as molecular signatures associated with the cross-talk between colorectal cancer and adipocytes. These molecules are associated with fatty acid metabolism and may be relevant to energy metabolic processes in CRC; however, future studies are needed to confirm this association. While the exact influence of these molecules on CRC requires further clarification, these discoveries mark a significant advancement, laying the groundwork for subsequent inquiries in this field.

## Data Availability

The raw data supporting the conclusions of this article will be made available by the authors, without undue reservation.
