# Supplementary material for: Metabolomics of 3D cell co-culture reveals alterations in energy metabolism at the cross-talk of colorectal cancer-adipocytes
Source: Front Med (Lausanne). 2024 Oct 3;11:1436866. doi: 10.3389/fmed.2024.1436866 (PMC11484090; doi:10.3389/fmed.2024.1436866)
Supplement: Supplementary file 1 [file Data_Sheet_1.DOCX]

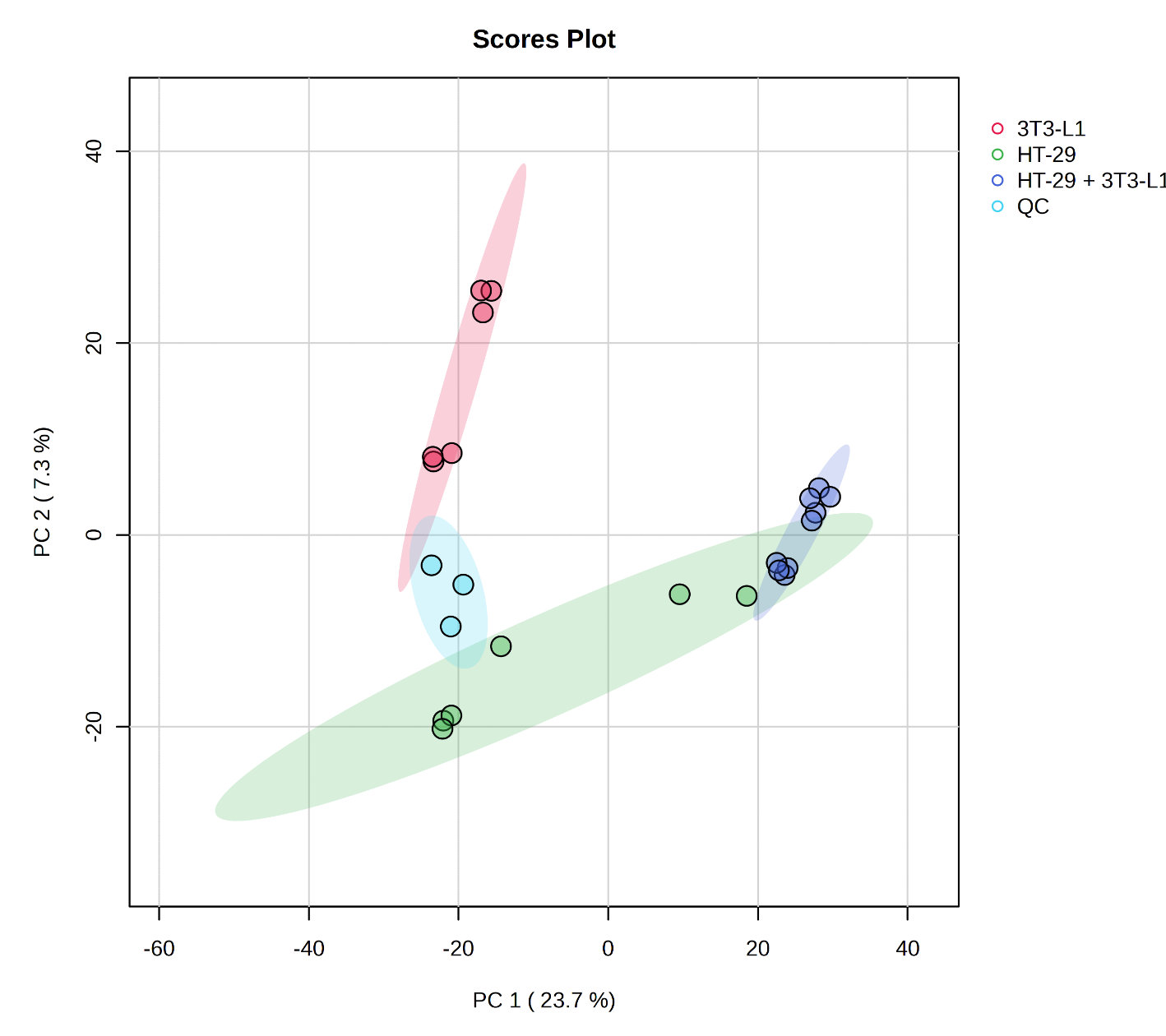


**Supplementary Figure**. The analytical quality and the liquid-chromatography coupled with high-resolution mass spectrometry reproducibility achieved by the clustering of the quality control (QC) samples in Principal Component Analysis scores plot; **3T3-L1** - pre-adipocytes cells; **HT-29** - human colon carcinoma; **3T3-L1 + HT-29** – three-dimensional co-culture.
