# Supplementary material for: Metabolomics of 3D cell co-culture reveals alterations in energy metabolism at the cross-talk of colorectal cancer-adipocytes
Source: Front Med (Lausanne). 2024 Oct 3;11:1436866. doi: 10.3389/fmed.2024.1436866 (PMC11484090; doi:10.3389/fmed.2024.1436866)
Supplement: Supplementary file 2 [file Data_Sheet_2.DOCX]

**Supplementary Table 1.** Features statistically different between groups. The False Discovery Rate (FDR) presented in the table refers to the ANOVA’s main effect.

| **m/z** | **FDR** |
| --- | --- |
| 0,50_255,0877m/z | 0.000094 |
| 0,58_665,2502m/z | 0.000094 |
| 5,23_478,2228m/z | 0.000096 |
| 5,61_423,0881m/z | 0.000133 |
| 0,73_257,9921m/z | 0.000133 |
| 7,39_421,2260m/z | 0.000145 |
| 1,63_339,1528m/z | 0.000160 |
| 0,53_974,4925m/z | 0.000168 |
| 1,16_508,0713m/z | 0.000168 |
| 1,63_417,0942m/z | 0.000168 |
| 0,84_170,8696m/z | 0.000173 |
| 0,56_510,2019n | 0.000173 |
| 10,73_515,2023n | 0.000194 |
| 0,53_724,8718m/z | 0.000195 |
| 1,17_679,1669n | 0.000209 |
| 1,17_1080,2085m/z | 0.000227 |
| 6,52_470,1909m/z | 0.000235 |
| 10,09_371,8686m/z | 0.000239 |
| 0,53_1292,4260n | 0.000248 |
| 10,11_124,0004m/z | 0.000278 |
| 4,84_380,0306m/z | 0.000299 |
| 5,84_437,1056m/z | 0.000319 |
| 0,53_1676,6958m/z | 0.000343 |
| 9,90_313,0774m/z | 0.000371 |
| 5,35_369,2281m/z | 0.000428 |
| 0,53_1138,4557m/z | 0.000428 |
| 0,87_335,0775m/z | 0.000492 |
| 0,91_242,0888n | 0.000494 |
| 5,48_396,0275m/z | 0.000494 |
| 11,01_632,3587m/z | 0.000494 |
| 0,77_84,0456m/z | 0.000503 |
| 0,86_477,1708m/z | 0.000626 |
| 0,53_1178,1263m/z | 0.000710 |
| 10,07_173,9771m/z | 0.000743 |
| 0,63_283,1008m/z | 0.001136 |
| 0,53_1631,7064m/z | 0.001201 |
| 1,56_339,0571m/z | 0.001243 |
| 0,54_677,6097m/z | 0.001243 |
| 1,19_198,0757m/z | 0.001259 |
| 7,55_504,2221m/z | 0.001426 |
| 0,56_571,1133m/z | 0.001426 |
| 5,42_413,2005m/z | 0.001562 |
| 1,17_715,1075m/z | 0.001890 |
| 9,12_610,3895m/z | 0.001890 |
| 1,63_288,0393m/z | 0.002283 |
| 0,53_1382,4259m/z | 0.002310 |
| 10,12_350,8596m/z | 0.002401 |
| 6,06_381,1893m/z | 0.002801 |
| 0,53_1246,4506m/z | 0.003209 |
| 0,53_1450,0783m/z | 0.003236 |
| 0,58_155,8649m/z | 0.003648 |
| 0,56_533,0721m/z | 0.003960 |
| 4,33_489,1880m/z | 0.004027 |
| 0,61_204,9572n | 0.004358 |
| 0,53_1563,3887m/z | 0.004358 |
| 6,48_390,2373m/z | 0.004417 |
| 5,83_220,1327m/z | 0.004692 |
| 7,15_520,3099m/z | 0.004692 |
| 8,36_412,2157m/z | 0.005050 |
| 1,17_1002,2665m/z | 0.005080 |
| 7,47_311,2588m/z | 0.005245 |
| 8,36_283,2423m/z | 0.005427 |
| 7,83_333,2182m/z | 0.007752 |
| 0,59_170,8695m/z | 0.007863 |
| 8,52_253,2161m/z | 0.009992 |
| 8,03_339,1991m/z | 0.010310 |
| 0,53_1235,2842m/z | 0.011498 |
| 0,53_1178,4592m/z | 0.012587 |
| 3,85_240,0824m/z | 0.012587 |
| 9,12_788,3833m/z | 0.014702 |
| 4,51_463,1127m/z | 0.020462 |
| 0,50_407,0741m/z | 0.020832 |
| 0,53_485,0339m/z | 0.021873 |
| 0,86_255,0475m/z | 0.021873 |
| 0,50_408,2332m/z | 0.023725 |
| 5,83_465,1528m/z | 0.024090 |
| 0,53_906,1721m/z | 0.024225 |
| 10,22_346,8900m/z | 0.025105 |
| 3,50_345,2022m/z | 0.025284 |
| 5,81_307,0536m/z | 0.025435 |
| 0,53_1269,1106m/z | 0.025435 |
| 8,86_505,3239m/z | 0.026378 |
| 1,17_479,0660n | 0.026378 |
| 0,53_759,8632n | 0.026378 |
| 0,58_153,8671m/z | 0.026717 |
| 4,58_267,0906m/z | 0.031106 |
| 9,25_485,2086m/z | 0.031206 |
| 0,63_288,9598m/z | 0.033028 |
| 9,07_313,2373m/z | 0.035721 |
| 0,53_1450,4153m/z | 0.036963 |
| 0,50_213,0841m/z | 0.036963 |
| 10,04_1017,2660m/z | 0.036963 |
| 1,17_481,0999m/z | 0.039841 |
| 0,77_268,0790n | 0.042641 |
| 0,53_1654,0339m/z | 0.042641 |
| 7,55_397,2255m/z | 0.042641 |
| 6,90_357,2265m/z | 0.042800 |
| 10,94_599,3184m/z | 0.043943 |
| 8,63_427,1629m/z | 0.044706 |
| 0,54_689,8399n | 0.044739 |
| 0,58_170,0450m/z | 0.047701 |
| 0,53_180,9716m/z | 0.048583 |
| 0,53_851,0494m/z | 0.049420 |
| 7,26_643,3305m/z | 0.049420 |
| 0,81_210,9737m/z | 0.049574 |
