# Supplementary material for: Metabolomics of 3D cell co-culture reveals alterations in energy metabolism at the cross-talk of colorectal cancer-adipocytes
Source: Front Med (Lausanne). 2024 Oct 3;11:1436866. doi: 10.3389/fmed.2024.1436866 (PMC11484090; doi:10.3389/fmed.2024.1436866)
Supplement: Supplementary file 3 [file Data_Sheet_3.DOCX]

**Supplementary Table 2.** Biological roles and process of the molecules highlighted in this study.

| **Molecule** | **Biological roles or process** |
| --- | --- |
| **9,10-Dihydroxy-**  **octadecenoic acid**  (C_18_H_34_O_4_) | - Apoptosis - Cholesterol translocation - Stabilizing cytochrome oxidase - Proton trap for oxidative phosphorylation - Stabilizing mitochondrial structure |
| **3-Hydroxytetracosanoyl-CoA**  (C_45_H_82_N_7_O_18_P_3_S) | - Membrane constituent - Fatty Acid Biosynthesis |
| **Palmitoleic acid**  (C_16_H_30_O_2_) | - Inner Membrane Transport - Fatty Acid Metabolism - Enzyme inhibitor (e.g. 5alpha-reductase inhibitor) |
| **Octadecenal**  (C_18_H_34_O) | - Lipid peroxidation - Fatty acid metabolismo - Cell signaling - Lipid metabolism pathway |
| **PA 18:4**  (C_21_H_33_O_8_P) | - Endocytosis - Apoptosis - Membrane Transport |
| **PG 20:0**  (C_26_H_51_O_10_P) | - Endocytosis - Apoptosis - Membrane Transport |
| **Acetylglutamic acid**  (C_7_H_11_NO_5_) | - Arginine Metabolism - Proline Metabolism - Ornithine Metabolism |

Data obtainned from Human Metabolome Database (HMDB) and LipidMaps databases, but other roles and processes to these molecules may be attributed.
